# A systematic review on the birth prevalence of metachromatic leukodystrophy

**DOI:** 10.1186/s13023-024-03044-w

**Published:** 2024-02-21

**Authors:** Shun-Chiao Chang, Aurore Bergamasco, Mélanie Bonnin, Teigna Arredondo Bisonó, Yola Moride

**Affiliations:** 1 Takeda Development Center Americas, Inc., Lexington, MA USA; 2 YOLARX Consultants, SAS, Paris, France; 3 YOLARX Consultants, Inc, Montreal, QC Canada

**Keywords:** Arylsulfatase A, Birth prevalence, Epidemiology, Lysosomal storage disease, Metachromatic leukodystrophy, Systematic review

## Abstract

**Background:**

Metachromatic leukodystrophy (MLD) is an autosomal recessive lysosomal storage disease caused by deficiency in arylsulfatase A (ASA) activity arising primarily from ASA gene (*ARSA*) variants. Late-infantile, juvenile and adult clinical subtypes are defined by symptom onset at ≤ 2.5, > 2.5 to < 16 and ≥ 16 years, respectively. Epidemiological data were sought to address knowledge gaps and to inform decisions regarding the clinical development of an investigational drug.

**Methods:**

To synthesize all available estimates of MLD incidence and birth prevalence worldwide and in selected countries, Ovid MEDLINE and Embase were searched systematically (March 11, 2022) using a population, intervention, comparator, outcome, time and setting framework, complemented by pragmatic searching to reduce publication bias. Where possible, results were stratified by clinical subtype. Data were extracted from non-interventional studies (clinical trials, non-clinical studies and case reports were excluded; reviews were used for snowballing only).

**Results:**

Of the 31 studies included, 14 reported birth prevalence (13 countries in Asia–Pacific, Europe, the Middle East, North America and South America), one reported prevalence and none reported incidence. Birth prevalence per 100,000 live births ranged from 0.16 (Japan) to 1.85 (Portugal). In the three European studies with estimates stratified by clinical subtypes, birth prevalence was highest for late-infantile cases (0.31–1.12 per 100,000 live births). The distribution of clinical subtypes reported in cases diagnosed over various time periods in 17 studies varied substantially, but late-infantile and juvenile MLD accounted for at least two-thirds of cases in most studies.

**Conclusions:**

This review provides a foundation for further analysis of the regional epidemiology of MLD. Data gaps indicate the need for better global coverage, increased use of epidemiological measures (e.g. prevalence estimates) and more stratification of outcomes by clinical and genetic disease subtype.

**Supplementary Information:**

The online version contains supplementary material available at 10.1186/s13023-024-03044-w.

## Background

Metachromatic leukodystrophy (MLD; OMIM 250100) is an inherited autosomal recessive lysosomal storage disease (LSD), usually caused by variants in the arylsulfatase A gene (*ARSA*), which encodes the enzyme arylsulfatase A (ASA) [1, 2]. Deficiency in ASA activity results in the accumulation of sulfatides in the central and peripheral nervous systems, leading to demyelination, neuronal dysfunction and degeneration [2, 3, 4]. This pathology manifests as motor and cognitive/behavioral impairments, usually culminating in profound disability and premature death [5]. The desulfation of sulfatides requires the combined action of ASA and its activator protein, saposin B; rarely, MLD may also be caused by a saposin B deficiency [6].

At least 200 pathogenic *ARSA* variants have been described [6, 7, 8, 9]. Some of these, such as the splice donor site variants c.465 + 1G > A (the most common variant in late-infantile MLD), result in the production of inactive ASA. Other variants, such as the missense variants c.1283C > T (the most common variant in adult MLD), result in ASA that retains some residual activity. Atypical MLD due to saposin B deficiency is caused by pathogenic variants in the prosaposin gene [10].

MLD is typically classified into three clinical subtypes [11, 12]. The most common subtype (approximately 50–60% of cases) is late-infantile MLD, which is characterized by symptom onset at age ≤ 2.5 years and is generally associated with the most rapid and severe disease progression. Juvenile MLD is defined by symptom onset at age > 2.5– < 16 years and typically represents 20–30% of cases; this subtype may be further subdivided into early-juvenile MLD (age at onset, > 2.5– < 6 years) and late-juvenile MLD (onset at age 6– < 16 years). Adult MLD (age at onset, ≥ 16 years) accounts for approximately 15–20% of cases. The types of first symptom (motor, cognitive or a combination of the two) have been associated with the rate of disease progression in MLD, along with age of onset [12]. Rapid disease progression has been associated with initial symptoms being predominantly motor in nature [12]. Decline in motor function in patients with MLD can be measured using the Gross Motor Function Classification in MLD [13], a standardized and disease-specific assessment tool that defines seven levels of capabilities in walking, sitting, and head and trunk control. The assessment of the regression of cognitive abilities varies across studies, and may involve the analysis of the loss of academic skills, such as concentration or reading/writing ability [12], or more standardized measures for intelligence quotients, such as the Wechsler Intelligence Scale for Children [14].

A gene therapy – atidarsagene autotemcel (Libmeldy, Orchard Therapeutics, Netherlands) – has been approved by the European Medicines Agency for the treatment of patients with late-infantile or early-juvenile MLD who have not yet developed symptoms, or patients with early-juvenile MLD who have initial symptoms but can still walk independently and before the onset of cognitive decline [15]. Other gene therapy and gene–cell therapy approaches are being explored [4], as well as intrathecally administered recombinant human ASA as a potential enzyme replacement therapy for MLD [16]. Hematopoietic stem cell transplantation has also been explored as a treatment option; however, the clinical benefit remains unclear and, in particular, treatment was associated with accelerated disease progression in some symptomatic patients. For symptomatic patients and those who do not meet specific criteria, the only current options available are to manage their existing symptoms.

To support the development and authorization of new therapeutics for MLD, it is important to understand the size, distribution and characteristics of the patient population. This is challenging, partly because difficulties in diagnosis and uncertainties about the pathogenic significance of some gene sequence variants may contribute to either under- or over-ascertainment of cases [17, 18]. It is typical to see birth prevalence ranges of 1.4–1.8 per 100,000 [7] or 1 in 40,000 to 1 in 160,000 [4] quoted in the literature; however, it is important to recognize regional and societal variations in the epidemiology of MLD that are biologically driven rather than a result of testing methodologies. For example, relatively high estimates of prevalence have been reported in regions such as the western Navajo Nation (1 in 2500 live births [19]) and in Arab groups of Israel (1 in 8000 live births [17]).

The objective of this systematic review was to generate a qualitative synthesis of estimates of incidence, birth prevalence and prevalence of MLD in countries across the world, stratifying results by clinical subtype.

## Methods

### Overview of the systematic review

A systematic review was conducted, following a population, intervention, comparator, outcomes, time and setting framework [20]. The population consisted of patients with MLD who received no interventions or standard of care, and studies were required to have either no comparator interventions or a comparator that was the standard of care. The outcomes of interest were the incidence, birth prevalence and prevalence of MLD in the general population (including all age and ethnic groups and both sexes) and the number of patients with MLD overall and according to clinical subtype. There was no restriction on the time period in which the observations were made or on the date of their report. The setting for studies reviewed was observational (non-interventional). The findings of the systematic review are reported according to the 2020 Preferred Reporting Items for Systematic Reviews and Meta-Analyses (PRISMA) statement [21].

### Data sources

Literature searches were conducted in the Ovid MEDLINE and EMBASE electronic bibliographical databases to cover the period from database inception (1946 and 1974, respectively) to the last search on March 11, 2022. The search terms are detailed in Additional file 2: Table S1. To identify additional relevant sources or references not indexed in these databases (e.g. some conference abstracts or recent publications) or indexed with keywords different from those defined in the search strategies, pragmatic web searches were conducted in English, French, Italian, Portuguese and Spanish using the search engines Google (www.google.com) and Google Scholar (www.scholar.google.com). These searches were complemented by hand searches of the websites for existing MLD registries (MLD Patient Powered Registry™ and the MLD initiative) and for a range of learned and clinical societies, patient associations, non-profit organizations, scientific conferences and rare diseases databases (Orphanet and the National Organization for Rare Diseases) (Additional file 2: Table S2). The same eligibility criteria used for full-text articles were applied for conference abstracts. Hand searches (referred to as snowballing) were also made of the reference lists of retained publications and of reviews (systematic or non-systematic) and meta-analyses identified by literature searches.

### Screening and full-text review

Duplicate publications in the search results returned from the bibliographic databases were removed, and the titles and/or abstracts of all unique sources were screened against predefined eligibility criteria (Table 1). Reviews and meta-analyses were used for snowballing only, and interventional clinical trials (phases 1–3) were excluded. The articles retained after screening and the sources identified by pragmatic searches and snowballing underwent an in-depth review of the full text to confirm eligibility. Screening and full-text review were conducted by two independent reviewers, with conflicts resolved by consensus or by a third independent reviewer (a senior epidemiologist).

**Table 1:** Eligibility criteria for inclusion of all sources (full-text articles and conference abstracts)

Inclusion criteria	Exclusion criteria
Studies conducted in humans Non-interventional (observational) studies (e.g. cohort studies, cross-sectional studies, case series, registries) Studies that included patients with MLD either as the study population or as a subgroup analysis Studies that reported on outcomes of interest (PICOTS) Original research articles published as full text or conference proceedings (i.e. posters, abstracts) Reviews (systematic or non-systematic) and meta-analyses (for snowballing only) For studies with multiple publications, only the latest publication on each outcome was retained Publications written in English, French, Italian, Portuguese or Spanish	Editorials, letters to the editors or opinions Case reports Clinical trials (phases 1–3) Non-clinical and experimental studies Studies reporting preliminary results (if later published as full text)

*MLD* metachromatic leukodystrophy; *PICOTS* population, intervention, comparator, outcomes, time and setting

### Data extraction

Two assessors who were working independently extracted the study characteristics and relevant data from the final selection of sources using a standardized data extraction form. Any conflict was resolved by consensus or by a third independent reviewer (a senior epidemiologist). The mode of source identification (literature search, pragmatic search or snowballing), the study reference, the type of source and the geographic coverage were recorded as general information. Specific information was extracted on the study methods (study period, study design, data collection method, name of source/center/study), the target population (MLD clinical subtype, target age group, specificities of target population) and the study population (inclusion/exclusion criteria, diagnostic criteria, sample size, distribution of cases according to MLD clinical subtype). Study results recorded were the duration of follow-up, patient characteristics, definition of the reference population, estimates of incidence and/or prevalence of MLD and distribution of prevalent MLD cases according to clinical subtype.

### Outcome definitions

For the purpose of this systematic review, birth prevalence was defined as the number of new cases of MLD diagnosed over a given time period divided by the number of live births within the same time period (also referred to as the lifetime risk of MLD at birth). The incidence (defined as the number of new MLD cases diagnosed over a specific time period) and prevalence (defined as the proportion of people alive on a certain date who had a diagnosis of MLD within a specified time period) were also investigated; however, no data on incidence were obtained from publications, and only one paper on prevalence was identified.

### Adherence to guidelines for systematic reviews

**Fig. 1:**
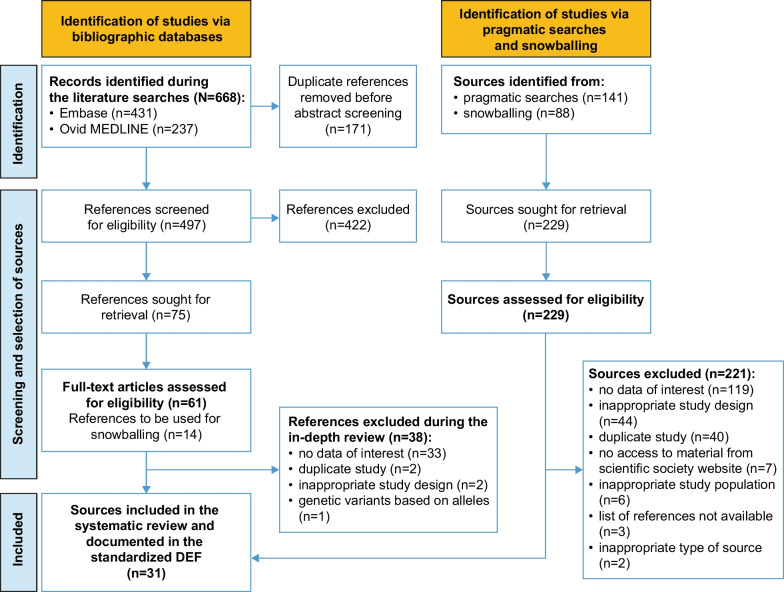
PRISMA flow chart of the systematic review process. *DEF* data extraction form; *PRISMA* Preferred Reporting Items for Systematic Reviews and Meta-Analysis

The systematic review protocol was registered in PROSPERO before the initiation of data extraction (PROSPERO registration: CRD42022320266). The principles of objectivity and transparency integral to a scientifically valid literature review were followed, and the review was conducted using the methods proposed by the Cochrane group [22] and the Institute of Medicine of the National Academy of Medicine [23]. All steps of the search process were documented and are presented in a PRISMA flow chart (Fig. 1) [21].

The methodological quality of studies reported in the retained full-text publications was assessed using the JBI checklist for studies reporting prevalence data [24], with the exception of conference abstracts, because they provide insufficient information for this process. Study quality was categorized by the number of the nine JBI criteria met: good = 7–9, moderate = 4–6 and low = 0–3. The study methodologies reported in full-text publications were also critically reviewed using the Guidelines for Good Pharmacoepidemiology Practices from the International Society for Pharmacoepidemiology as a framework [25]. The main strengths and limitations/risk of bias of individual studies were summarized in the data extraction form; these results were used to interpret results from individual studies. Methodological quality assessment was not performed for conference abstracts because the information provided in these types of publications is insufficient for such assessments.

## Results

### Summary of search results

The literature search identified 668 references, of which 497 were unique and were manually screened for eligibility (Fig. 1). After abstract screening, 75 references were retained for full-text review to confirm eligibility (61 original studies, 14 literature reviews used for snowballing only [retention rate: 15.1%]). The agreement rate between the two assessors was 92.2%. During the in-depth review of the 61 original studies, 38 were further excluded (mostly owing to not reporting incidence or prevalence [n = 33]; see Fig. 1 for further details). Pragmatic searches identified two additional sources; snowballing identified a further six sources. In total, 31 sources were included in this systematic review and underwent data extraction, including three conference proceedings [26, 27, 28].

Of the 28 full publications, six were rated as good quality [29, 30, 31, 32, 33, 34], 17 as moderate quality [9, 12, 35, 36, 37, 38, 39, 40, 41, 42, 43, 44, 45, 46, 47, 48, 49] and five were of low quality [50, 51, 52, 53, 54] (Additional file 1: Fig. S1). Among the studies of moderate or low quality (n = 22), the main methodological gaps were insufficient information on the response rate and statistical analysis, limited coverage of the identified sample (i.e. poor generalizability of estimates) and lack of details on study participants and setting.

### Birth prevalence of MLD

**Fig. 2:**
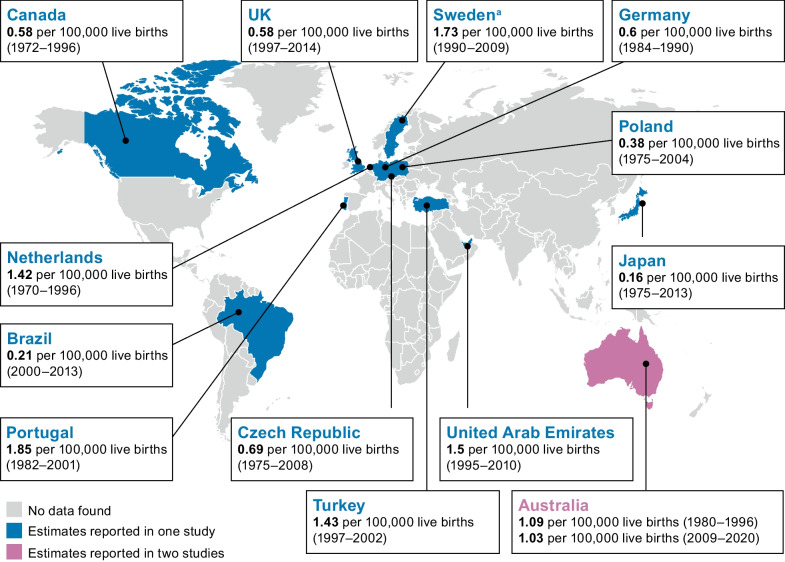
Regional birth prevalence of MLD. Birth prevalence refers to the number of new MLD diagnoses (prenatal and postnatal) divided by the number of live births over a specified period. ^a^Only postnatal diagnoses were considered; prenatal diagnoses were excluded owing to a lack of complete data. *MLD* metachromatic leukodystrophy

In total, 14 studies from 13 countries reported birth prevalence of MLD: 10 retrospective cohort studies [29, 31, 32, 33, 34, 37, 41, 43, 44, 46], three cross-sectional studies [30, 38, 39] and one prospective cohort study [48]. Each study reported data for a single country, with two studies reporting data from Australia. Table 2 provides summary information on these studies, along with case identification/diagnostic criteria, if reported. Most of these studies (12/14) [29, 30, 31, 32, 33, 34, 37, 38, 39, 41, 44, 46]) covered all age groups, but the remaining two were specific to the pediatric population (age < 16 years) [43, 48]. All studies included both prenatal and postnatal diagnoses of MLD, except for one study from Sweden, which excluded prenatal diagnoses owing to lack of complete data [32]. A graphical representation of reported estimates by geographic region is presented in Fig. 2. Reported estimates ranged from 0.16 to 1.85 per 100,000 live births.

**Table 2:** Studies reporting estimates of birth prevalence of MLD

Reference/study design (quality^a^)	Country	Sample size	MLD case identification method/diagnostic criteria	Birth prevalence (per 100,000 live births)
*Europe*				
Ługowska et al. [41]/retrospective cohort (moderate)	Poland	60	• Based on diagnosed cases, but diagnostic tests not reported	0.38
			• Expected prevalence of conceived fetuses with two pathogenic *ARSA* variants based on population carrier rates	4.1 (95% CI: 1.8–9.4)
Heim et al. [38]/cross-sectional (moderate)	Germany	125	• Strongly reduced ASA activity in leukocytes or cultured skin fibroblasts (specific ASA activity level considered to be not specified) • In the case of multiple sulfatase deficiency, presence of a deficiency of several different sulfatases in fibroblasts or urine • Clinical characteristics – Typical age at onset, development of spastic tetraparesis, incontinence, optic atrophy and peripheral neuropathy in combination with CT- or MRI-proven white matter involvement	0.6
Poupětová et al. [46]/retrospective cohort (moderate)	Czech Republic	25	• Deficiency of the relevant enzyme • Presence of the pathogenic variation • Detection of undegraded substrate by loading tests in cell cultures	All MLD (N = 25): 0.69 (95% CI: 0.29–1.38) – Late-infantile (n = 13): 0.31 – Juvenile (n = 5): 0.11 – Adult (n = 6): 0.27
Poorthuis et al. [34]/retrospective cohort (good)	Netherlands	103	• Not specified	All MLD (N = 103): 1.42 – Late-infantile (n = 28): 0.52 – Juvenile (n = 41): 0.51 – Adult (n = 23): 0.24 – Unspecified (n = 11): 0.15
Hult et al. [32]/retrospective cohort (good)	Sweden	47	• Quantitative and qualitative determination of urinary glycosaminoglycans and oligosaccharides • Determination of enzyme activities	1.73 (excluding prenatal diagnosis)
Pinto et al. [44]/retrospective cohort (moderate)	Portugal	21	• Enzymatic activity determined in a blood sample and subsequently confirmed in cultured skin fibroblasts • Urinary excretion of substrates • Genotype analysis	All MLD (N = 21): 1.85 – Late-infantile (n = 11): 1.12 – Juvenile (n = 2): 0.29 – Adult (n = 7): 0.45
Stellitano et al. [48]/prospective cohort (pediatric population) (moderate)	UK	76	• Not specified	0.58
*North America*				
Applegarth et al. [30]/cross-sectional (good)	Canada	6	• Appropriate criteria for diagnosis of a genetically inherited metabolic disease included appropriate family studies of the metabolic defect • Laboratory tests – Quantitative plasma and CSF amino acid analyses – Urine organic acids by gas chromatography–mass spectrometry – Specific enzyme assays – Prenatal diagnosis	0.58
*Asia–Pacific*				
Koto et al. [39]/cross-sectional (moderate)	Japan	24	• For late-infantile MLD (representing most part of sample size): – enzyme activity test (86.7%^b^) – genetic testing (53.3%^b^)	0.16
Chin et al. [31]/retrospective cohort (good)	Australia	38	• Biochemical assessments – Deficient enzyme – Elevated substrate biomarkers • Molecular genetic testing that identified pathogenic variants	All MLD: 1.03 – Postnatal: 1.00
Meikle et al. [33]/retrospective cohort (good)	Australia	46	• Enzymatic analysis	All MLD: 1.09 (equivalent to 1 per 92,000, as per publication) – Postnatal: 0.83 (equivalent to 1 per 121,000, as per publication)
*South America*				
Giugliani et al. [37]/retrospective cohort (moderate)	Brazil	150	• Quantitation and electrophoresis of urinary glycosaminoglycans • Specific fluorometric, colorimetric or radio isotopic enzyme assays and/or by identification of pathogenic variations in blood or fibroblasts cultivated from skin biopsies	0.21
*Middle East*				
Al-Jasmi et al. [29]/retrospective cohort (good)	United Arab Emirates	3	• Clinical presentation • Biochemical analysis performed at two referral centers	1.5
Ozkara et al. [43]/retrospective cohort (pediatric population) (moderate)	Turkey	93	• Enzyme deficiency – Postnatal diagnosis: leukocytes sample – Prenatal diagnosis: chorionic villus, amnion cell culture and cord blood samples	1.43

*ARSA* arylsulfatase A gene; *ASA* arylsulfatase A; *CI* confidence interval; *CSF* cerebrospinal fluid; *CT* computed tomography; *MLD* metachromatic leukodystrophy; *MRI* magnetic resonance imaging

^a^Based on the JBI checklist. Good methodological quality: 7–9 items met on the JBI checklist; moderate methodological quality: 4–6 items met on the JBI checklist; low methodological quality: 0–3 items met on the JBI checklist

^b^Percentages represent the proportion of patients who received a diagnosis with each method

Estimates of birth prevalence per 100,000 live births in European countries (seven studies; seven countries) [32, 34, 38, 41, 44, 46, 48] ranged from 0.38 in a retrospective cohort study in Poland between 1975 and 2004 [41] to 1.85 in a retrospective cohort study conducted between 1982 and 2001 in the Northern Portuguese population [44] (Table 2). The Polish study also reported MLD birth prevalence based on carrier rates of pathogenic *ARSA* variants: this was 4.1 (95% confidence interval [CI]: 1.8–9.4) per 100,000 live births [41]. Three European studies reported birth prevalence per 100,000 births both overall and by MLD clinical subtype (Table 2) [34, 44, 46]. These estimates for all MLD, late-infantile MLD, juvenile MLD and adult MLD subtypes were 0.69, 0.31, 0.11 and 0.27, respectively, in the Czech Republic between 1975 and 2008 [46], 1.42, 0.52, 0.51 and 0.24, respectively, in the Netherlands between 1970 and 1996 (0.15 for unspecified subtypes) [34], and 1.85, 1.12, 0.29 and 0.45, respectively, in Portugal for periods defined by the birth dates of the cases [44]. Birth prevalence of MLD in the UK was estimated only for a pediatric population (age < 16 years) sourced from the British Paediatric Surveillance Unit system between 1997 and 2014, and was equivalent to 0.58 per 100,000 live births [48].

One study reported birth prevalence data from North America [30]. In British Columbia in Canada, six patients received a diagnosis of MLD at a central referral center for metabolic disorders in this region between 1972 and 1996. The British Columbia Vital Statistics Agency reported 1,035,816 births during the same period, so the estimated birth prevalence of MLD was 0.58 per 100,000 live births.

There were three Asia–Pacific studies with birth prevalence data [31, 33, 39]; two of these were studies conducted in Australia that used the same data source (the National Referral Laboratory for LSD diagnoses in Australia) over different time periods [31, 33]. There were similar prevalence estimates between studies for prenatal and postnatal diagnoses combined: 1.09 per 100,000 live births between 1980 and 1996 [33] and 1.03 per 100,000 live births between 2009 and 2020 [31]. Estimates were also similar between studies when only postnatal cases were counted, being 0.83 per 100,000 live births (equivalent to the 1.00 per 121,000 reported) in the earlier study [33] and 1.00 per 100,000 live births in the other study [31]. The study from Japan in patients treated between 2013 and 2016 reported a birth prevalence of 0.16 per 100,000 live births, which was the lowest rate identified in this systematic review [39].

There was one study from South America with birth prevalence data – a retrospective cohort study based on the medical records of the Hospital de Clinicas de Porto Alegre in Brazil [37]. This study reported a relatively low estimated birth prevalence of 0.21 per 100,000 live births for the period between 2000 and 2013 [37].

The two studies from two countries in the Middle East (the United Arab Emirates and Turkey) reported similar estimates of birth prevalence of MLD [29, 43]. One was a retrospective cohort study in the only two metabolic referral centers in the United Arab Emirates that estimated the birth prevalence of MLD to be 1.5 per 100,000 live births between 1995 and 2010 [29]. The other study was a retrospective cohort study of medical records from a Turkish children’s hospital and reported an estimate of 1.43 cases of MLD per 100,000 live births, based on a pediatric population (all late-infantile MLD, age < 5 years) between 1997 and 2002 [43].

For context, we provide estimates of the number of live births for each country covered in this review for 2023 (Additional file 2: Table S3 [55]).

The prevalence of MLD was reported in one retrospective cohort study conducted in a pediatric population (age ≤ 18 years) in the USA [36]. The 2‑year point prevalence in different ethnic subgroups was estimated by a generalized linear model using data from pediatric patients admitted to a Pediatric Health Information System hospital between October 2015 and September 2017. In total, 139 patients were identified as having MLD. The reported crude 2‑year prevalence of MLD (95% CI) per 100,000 patients was significantly higher in patients of American Indian ethnicity (13.9 [3.5–55.4]; *p* = 0.01) and significantly lower in patients of white Hispanic ethnicity (0.9 [0.5–1.8]; *p* = 0.02) than in white non-Hispanic patients (2.2 [1.7–2.8]) (Table 3).

**Table 3:** Estimated 2-year point prevalence of MLD according to race and ethnicity

Race and/or ethnicity	Unadjusted^a^ prevalence per 100,000 patients (95% CI)	*p* value
White non-Hispanic	2.2 (1.7–2.8)	Reference
Asian	2 (0.6–6.1)	0.85
Black Hispanic	0 (0–100,000)	> 0.99
Black non-Hispanic	1.6 (1.0–2.5)	0.22
Multiple	1.4 (0.7–2.7)	0.20
American Indian	13.9 (3.5–55.4)	**0.01**
Other	4.1 (2.6–6.6)	**0.02**
Pacific Islander	0 (0–100,000)	> 0.99
White Hispanic	0.9 (0.5–1.8)	**0.02**

*CI* confidence interval; *MLD* metachromatic leukodystrophy

Bold values denote significance

Data are from a study of children and adolescents in the US Children’s Hospital Association’s Pediatric Health Information System database from October 2015 to September 2017; of these patients, 139 received a diagnosis of MLD [36]

^a^There were no adjustments between cases with and without MLD in the database to account for potential differences in sex, insurance type, urban or rural status, household income, number of inpatient days for the patient for all admissions during the study, or patient age

### Distribution of MLD cases

**Fig. 3:**
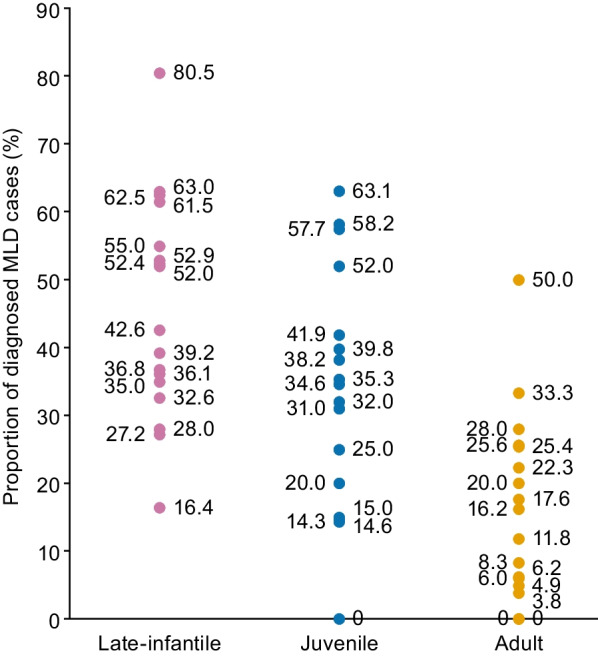
Distribution of MLD diagnoses according to clinical subtype. *MLD* metachromatic leukodystrophy

Although birth prevalence estimates stratified by MLD clinical subtype have been reported, 17 studies reported the distribution of MLD cases diagnosed over various time periods by clinical subtype [9, 12, 26, 34, 35, 38, 39, 40, 42, 44, 45, 46, 47, 51, 52, 53, 54] (Table 4). The proportions of each subtype varied considerably between studies, but the predominant subtype in 10/17 studies was late-infantile MLD, accounting for 39.2–80.5% of MLD cases identified [26, 38, 39, 42, 44, 45, 46, 47, 51, 52]; juvenile MLD was the predominant subtype in 6/17 studies (39.8–63.1% of recruited patients) [9, 12, 34, 35, 40, 54], and adult MLD was the least frequent clinical subtype in 13/17 studies (0–25.6% of MLD cases identified) [12, 26, 34, 35, 38, 39, 40, 42, 45, 47, 51, 52, 54] (Fig. 3). In the eight studies that subdivided juvenile MLD [9, 12, 26, 40, 47, 52, 53, 54], there was a clear difference in prevalence between the early- and late-juvenile forms; the dominant form was early-juvenile MLD in five of the studies [26, 40, 47, 52, 54]. Across the 17 studies, late-infantile and juvenile MLD together accounted for at least two-thirds of cases in most studies (Table 4). Adult MLD was the least frequently observed subtype in most studies, and also the most variable subtype between studies.

**Table 4:** Studies reporting distribution of MLD clinical subtypes in diagnosed MLD cases

Reference/study design (quality^a^)	Country/ies	Sample size	Distribution of MLD clinical subtype
*Europe*			
Heim et al. [38]/cross-sectional (moderate)	Germany	125	• Late-infantile: 39.2% (n = 49) • Juvenile: 32.0% (n = 40) • Adult: 17.6% (n = 22) • Unspecified: 10.4% (n = 13) Remaining case (n = 1) was MLD‑MSD
Poorthuis et al. [34]/retrospective cohort (good)	Netherlands	103	• Late-infantile: 27.2% (n = 28) • Juvenile: 39.8% (n = 41) • Adult: 22.3% (n = 23) • Unspecified: 10.7% (n = 11)
Kehrer et al. [12]/prospective cohort (moderate)	Germany	97	• Late-infantile: 36.1% (n = 35) • Juvenile: 57.7% (n = 56) – Early-juvenile: 32.1% (n = 18) – Late-juvenile: 67.8% (n = 38) • Adult: 6.2% (n = 6)
Polten et al. [45]/cross-sectional (moderate)	Germany	68	• Late-infantile: 42.6% (n = 29) • Juvenile: 38.2% (n = 26) • Adult: 16.2% (n = 11) • Unspecified: 2.9% (n = 2)
Beerepoot et al. [9]/retrospective cohort (moderate)	Netherlands	67	• Late-infantile: 16.4% (n = 11) • Juvenile: 58.2% (n = 39) – Early-juvenile: 35.9% (n = 14) – Late-juvenile: 64.1% (n = 25) • Adult: 25.4% (n = 17)
Ługowska et al. [40]/cross-sectional (moderate)	Poland	43	• Late-infantile: 32.6% (n = 14) • Juvenile: 41.9% (n = 18) – Early-juvenile: 61.1% (n = 11) – Late-juvenile: 38.9% (n = 7) • Adult: 25.6% (n = 11)
Berger et al. [35]/cross-sectional (moderate)	Austria, Croatia, Germany and Poland	27	Based on a total of 25 unrelated patients • Late-infantile: 28.0% (n = 7) • Juvenile: 52.0% (n = 13) • Adult: 20.0% (n = 5)
Biffi et al. [52]/prospective cohort (low)	Italy	26	• Late-infantile: 61.5% (n = 16) • Juvenile: 34.6% (n = 9) – Early-juvenile: 77.8% (n = 7) – Late-juvenile: 22.2% (n = 2) • Adult: 3.8% (n = 1)
Poupětová et al. [46]/retrospective cohort (moderate)	Czech Republic	25	• Late-infantile: 52.0% (n = 13) • Juvenile: 20.0% (n = 5) • Adult: 28.0% (n = 7)
Pinto et al. [44]/retrospective cohort (moderate)	Portugal	21	• Late-infantile: 52.4% (n = 11) • Juvenile: 14.3% (n = 3) • Adult: 33.3% (n = 7)
Barth et al. [51]/prospective cohort (low)	UK	17	• Late-infantile: 52.9% (n = 9) • Juvenile: 35.3% (n = 6) • Adult: 11.8% (n = 2)
*North America*			
Bascou et al. [26]^b^/prospective cohort	USA	122	• Late-infantile: 63% • Juvenile: 31% – Early-juvenile: 64.5% – Late-juvenile: 35.5% • Adult: 6%
*Asia–Pacific*			
Narayanan et al. [42]/cross-sectional (moderate)	India	41	• Late-infantile: 80.5% (n = 33) • Juvenile: 14.6% (n = 6) • Adult: 4.9% (n = 2)
Lomash et al. [27]^b^/prospective cohort	India	22	n numbers not reported for breakdown
Koto et al. [39]/cross-sectional (moderate)	Japan	24	• Late-infantile: 62.5% (n = 15) • Adult: 8.3% (n = 2) • Unknown: 29.2% (n = 7)
Hettiarachchi et al. [53]/prospective cohort (low)	Sri Lanka	20	• Late-infantile: 35.0% (n = 7) • Juvenile: 15.0% (n = 3) – Early-juvenile: 33.3% (n = 1) – Late-juvenile: 66.7% (n = 2) • Adult: 50.0% (n = 10)
Shukla et al. [47]/cross-sectional (moderate)	India	20	• Late-infantile: 55.0% (n = 11) • Juvenile: 25.0% (n = 5) – Early-juvenile: 80.0% (n = 4) – Late-juvenile: 20.0% (n = 1) • Adult: 0.0% (n = 0) • Asymptomatic^c^: 20.0% (n = 4)
*South America*			
Virgens et al. [49]/retrospective cohort (moderate)	Brazil	27	–
*Middle East*			
Mahdieh et al. [54]/retrospective cohort (low)	Iran	19	• Late-infantile: 36.8% (n = 7) • Juvenile: 63.1% (n = 12) – Early-juvenile: 66.7% (n = 8) – Late-juvenile: 33.3% (n = 4) • Adult: 0.0% (n = 0)
Al-Hassnan et al. [50]/retrospective cohort (low)	Saudi Arabia	16	–

*MLD* metachromatic leukodystrophy; *MLD-MSD* MLD-multiple sulfatase deficiency

^a^Based on the JBI checklist. Good methodological quality: 7–9 items met on the JBI checklist; moderate methodological quality: 4–6 items met on the JBI checklist; low methodological quality: 0–3 items met on the JBI checklist

^b^Reported as a conference abstract; insufficient information available for assessment of methodological quality

^c^Patients with positive family history and deficient ASA activity

## Discussion

This systematic review identified 31 sources, which reported estimates of birth prevalence of MLD (lifetime risk of MLD at birth) in nine countries covering Asia–Pacific, Europe, the Middle East, and North and South America. Additionally, the sources reported the distribution of diagnosed MLD cases according to clinical subtype in 12 countries, covering the same regions.

Estimates of the birth prevalence of MLD varied by a factor of 10 between the lowest and the highest values reported. This heterogeneity probably reflected a combination of genuine differences in prevalence (e.g. regional trends in the frequency of gene variants or consanguinity), methodological differences in case ascertainment (e.g. data source and confirmatory diagnostic tests) and differences in the way the birth prevalence was calculated. It is also possible that, within a study, factors that would tend to lead to underestimation of birth prevalence may have been balanced by factors that would otherwise lead to overestimation of birth prevalence.

The low estimate of birth prevalence in Japan (0.16 per 100,000 live births) is likely to have been an underestimate because it was based on the number of individuals who were treated within a 3‑year period divided by the total number of live births between the birth years of the oldest and youngest cases [39]. This would not have accounted for patients who had received a diagnosis during this birth period but who were not alive at the time of the survey. In addition, the correction for ‘double counting’ of patients appears to have been calculated from only a proportion of the hospitals that contributed cases (303 of 504 hospitals [60.1%]).

Similarly, the study of birth prevalence of LSDs (including MLD) in the Czech Republic [46] and a review of lifetime risk calculation methods for Krabbe disease [56] highlight the importance of the relationship between the chronological period used for the numerator and the one used for the denominator in the calculation. These periods were different in the Czech study because births in the general population for the denominator were taken from the period delineated by the year of birth of the oldest patient and the year of birth of the youngest patient (the birth period) among cases diagnosed in the predetermined period of data collection (the diagnosis period) [46]. As with the Japanese study, the extent of the underestimation of birth prevalence due to individuals with MLD being born during the birth period but having died before the diagnosis period depends partly on the duration of the diagnosis period and the distribution of clinical subtypes. The occurrence of adult MLD extends the birth period and, therefore, increases the potential for underestimation. The same effect might also result from delayed diagnoses caused by failure to recognize early clinical signs and symptoms and insufficient access to or uptake of prenatal screening.

The next lowest birth prevalence after Japan was in Brazil (0.21 per 100,000 live births), and this may have reflected depressed case ascertainment; the publication acknowledged that the outcome was a minimum value because, although the data were from the reference laboratory, logistical difficulties and regional deficiencies in health-care provisions meant that not all cases would have been referred [37]. The relatively low birth prevalence of MLD in Poland based on diagnosed cases was approximately tenfold lower than that anticipated from carrier rates of pathogenic *ARSA* variants [41]. The carrier rates were taken from a single study group with a sample size of only 60 individuals, and projections for the consequent number of homozygotes did not allow for prenatal or perinatal death of fetuses with a severe phenotype which would have decreased prevalence among live-born children. Nonetheless, preliminary newborn screening (NBS) data from Europe indicate that carrier rates may offer a more accurate indication of true MLD prevalence than the number of diagnosed cases [57].

Relatively high estimates of birth prevalence were reported in the Middle East (1.5 per 100,000 live births in the general population and 1.43 per 100,000 in the pediatric population), probably owing to higher consanguinity rates than in other countries [29, 43]. The highest estimate was reported by the study in Portugal, (1.85 per 100,000 live births), although the authors of that study noted that this may have been driven by the high frequency of the IVS2 + 1G > A *ARSA* variant in this population (60% in Portugal vs 15–43% in other European countries) [44]. IVS2 + 1G > A is a pathogenic splice donor site variant and results in a null allele unable to make any detectable functional ASA enzyme. As such, it is usually associated with severe symptoms and an early onset of MLD; this is consistent with the majority of patients in the Portuguese study having received a diagnosis of late-infantile MLD.

It was not possible to gauge whether the availability of prenatal diagnoses affected the birth prevalence data generally, but, at least in the context of the two Australian studies, it did not appear to have an effect [31, 33]. The only study to exclude prenatal diagnoses was in Sweden, and it reported one of the highest estimated birth prevalence rates (1.73 per 100,000) [32]. Another limitation of the review is that only four of the 11 studies that described the diagnostic inclusion criteria used genetic testing. This may have had a significant impact on the reported birth prevalence rates. Establishing a diagnosis of MLD is multifaceted and may include assessment of enzymatic activity, measuring levels of urinary excretion of substrates and magnetic resonance imaging evidence [17]; however, normal findings from these assessments do not necessarily exclude a diagnosis of MLD, and confirmation with genetic testing is advised [58]. Therefore, some studies included in this review may have reported an underestimation of the true birth prevalence.

The birth prevalence estimate by clinical subtype of MLD was highest for late-infantile cases in each of the three European studies reporting this breakdown [34, 44, 46]. In addition, late-infantile and juvenile MLD together tended to account for approximately two-thirds of cases across studies. Adult MLD was the least frequently observed subtype. No other clear trends were evident.

The single study reporting prevalence of MLD as distinct from birth prevalence presented the data stratified by patient ethnicity only, with no overall prevalence [36]. Significant differences in prevalence were observed between ethnic subgroups, and further investigation is warranted. The study was unable to determine whether these differences in prevalence were associated with socioeconomic or cultural impacts on diagnosis, or whether they were by driven by any differences between ethnic groups in the frequency of pathogenic *ARSA* alleles.

This systematic review highlights several data gaps that, if closed, could help to advance our understanding of the epidemiology of MLD. Data were absent or limited for many regions, including Africa, China and the USA. In addition, data were reported for birth prevalence only (or lifetime risk of MLD at birth; the number of new cases of MLD diagnosed over a given time period divided by the number of live births within the same time period); no data were identified on the incidence or prevalence of MLD. Data on incidence and prevalence, which derive the absolute number of new and existing cases of MLD, respectively, would be more informative for drug development than a ratio to number of live births.

This review benefited from a robust design and careful execution. The impact of publication bias was reduced by searching two complementary electronic bibliographical databases, coupled with pragmatic searches using search engines for gray literature. Data extraction from the selected publications was duplicated by two independent reviewers to ensure the consistency and accuracy of the data. JBI rating was used to assess the quality of the included studies, 82% of which were rated as being of high or moderate quality. The search strategy was comprehensive but, as with any literature review, there is the possibility that relevant studies were missed. All relevant data available from studies meeting the eligibility criteria were considered, regardless of the study sample size or other study limitations. The generalizability of some country-specific data may be limited by the small sample size (e.g. the only North American study included only six patients with MLD [30]). Differences in birth prevalence calculations may have resulted in some studies having a greater potential for underestimation than others.

Further research is needed to document MLD cases worldwide and to improve our understanding of the distribution of MLD cases by disease severity. At present, the total number of patients with MLD in countries of interest may need to be estimated indirectly using available data on regional birth prevalence rates and other population- and disease-related parameters. However, the introduction of disease registries may provide a rich database to answer epidemiological and clinical questions. Registries for mucopolysaccharidosis II and Fabry disease, for example, have improved our understanding of the epidemiology and the natural history of disease progression, as well as providing long-term data on the safety and effectiveness of treatments [61, 62]. The initiation of the international registry, the MLD initiative, in 2020 [63] is a positive step in improving epidemiological data in the MLD space.

Findings from this systematic review and associated future work have practical relevance in informing local decisions on the implementation of NBS for MLD. NBS for MLD using blood spots is now feasible and specific [64], and could change the landscape of MLD epidemiology. As well as improving patient outcomes, it has the potential to affect the reported incidence and/or reported prevalence of MLD in different countries at different times depending upon availability and uptake. NBS is likely to provide new insights into prevalence and new challenges in data interpretation. For example, when screening for MLD by quantification of ASA in dried blood spots via immunoassay, there is a risk of false-negative results due to the presence of conformationally normal ASA that is deficient in enzyme activity [64, 65]. Low ASA activity itself does not necessarily result in symptomatic disease, so it is recommended that NBS strategies combine measurement of ASA activity, quantification of sulfatide and genetic testing [66]. An ongoing study of a pilot scheme in Northern Germany, for example, has offered proof of concept of a high-throughput method for MLD NBS incorporating testing for ASA activity, sulfatide levels and genetic confirmation [57]. Prospective screening commenced in October 2021; three MLD screening-positive cases had been identified by January 2023 (one clinically confirmed, two pending clinical confirmation) from more than 81,000 babies screened. Improved understanding of biomarkers and the influence of genotype on MLD phenotype will be needed to support increased uptake of NBS for MLD. For example, a study by Santhanakumaran and colleagues found that the c.465 + 1G > A *ARSA* variant was most commonly associated with late-infantile MLD, the c.1283C > T *ARSA* variant with juvenile MLD and the c.542T > G *ARSA* variant with adult MLD [59]. It would be beneficial to investigate further epidemiological questions stratified by genetic variant as the field progresses.

## Conclusion

This systematic review has generated a robust summary of available data on the birth prevalence of MLD and has highlighted knowledge gaps, particularly with respect to incidence data. These findings set a foundation for further analysis of regional epidemiology of MLD, which will be important to support advances in MLD management (such as NBS) for improved diagnosis.

### Supplementary Information

**Additional file 1.** Supplementary Figure.

**Additional file 2.** Supplementary Tables.

## Data Availability

Data sharing is not applicable to this article because no new data sets were collected, generated or analyzed for this study. All data supporting the findings were publicly available at the time of submission and are summarized in the article (Tables 2 and 4, Figs. 1–3 and Additional file 2: Tables S1 and S2). Full data can be obtained from this article and the cited studies.

## Abbreviations

*ARSA*: Arylsulfatase A gene
ASA: Arylsulfatase A
CI: Confidence interval
LSD: Lysosomal storage disease
MLD: Metachromatic leukodystrophy
NBS: Newborn screening
PRISMA: Preferred Reporting Items for Systematic Reviews and Meta-Analyses
