# Supplementary material for: A systematic review on the birth prevalence of metachromatic leukodystrophy
Source: Orphanet J Rare Dis. 2024 Feb 21;19:80. doi: 10.1186/s13023-024-03044-w (PMC10880320; doi:10.1186/s13023-024-03044-w)
Supplement: Supplementary file 2 — Additional file 2. Supplementary Tables. [file 13023_2024_3044_MOESM2_ESM.docx]

**Supplementary Fig. 1** Ratings of the JBI checklist for 28 included studies

Three additional publications (conference proceedings) were not assessed owing to a lack of information reported

Supplementary Table 1 Literature search terms and results

| # | Searches | Number of hits |
| --- | --- | --- |
| MEDLINE | | |
| 1 | Leukodystrophy, metachromatic/ | 1282 |
| 2 | metachromatic leukodystrophy*.mp. | 1224 |
| 3 | 1 OR 2 | 1672 |
| 4 | exp Epidemiology/ or incidence/ or prevalence/ | 615 121 |
| 5 | (epidemiology or inciden* or prevalen* or gross motor function or GMFC-MLD).mp. | 3 166 667 |
| 6 | 4 OR 5 | 3 167 608 |
| 7 | exp genetic association studies/ | 66 395 |
| 8 | variant*.ab,ti. | 393 259 |
| 9 | 7 OR 8 | 436 447 |
| 10 | 6 OR 9 | 3 545 229 |
| 11 | **3 AND 10** | **237** |
| Embase | | |
| 1 | metachromatic leukodystrophy/ | 2209 |
| 2 | metachromatic leukodystrophy*.mp. | 2408 |
| 3 | 1 OR 2 | 2408 |
| 4 | exp Epidemiology/ or incidence/ or prevalence/ | 3 967 534 |
| 5 | (epidemiology or inciden* or prevalen* or gross motor function or GMFC-MLD).mp. | 3 692 891 |
| 6 | 4 OR 5 | 5 600 542 |
| 7 | exp genetic association study/ | 39 834 |
| 8 | variant.ab,ti. | 277 760 |
| 9 | 7 OR 8 | 313 090 |
| 10 | 6 OR 9 | 5 864 347 |
| 11 | **3 AND 10** | **431** |

*truncation symbol; ab,ti: abstract/title; mp: multipurpose (i.e. searching for a term without specifying a field). Date of last search for Embase and MEDLINE: March 11, 2022

Supplementary Table 2 Additional data sources (hand-searched)

| Category | Sources |
| --- | --- |
| Existing MLD registries | MLD Patient Powered Registry |
|  | The MLD Initiative |
| Learned/clinical societies | American College of Medical Genetics and Genomics (ACMG) |
|  | American Neurological Association (ANA) |
|  | Child Neurology Society (CNS) |
|  | European Academy of Neurology (EAN) |
|  | European Paediatric Neurology Society (EPNS) |
|  | European Reference Network-Rare Neurological Disease (ERN-RND) |
|  | European Society of Gene and Cell Therapy (ESGCT) |
|  | European Society of Human Genetics (ESHG) |
|  | Global Leukodystrophy Initiative Association (GLIA) |
|  | International Genetic Epidemiology Society (IGES) |
|  | International Society of Pharmacoeconomics and Outcomes Research (ISPOR) |
|  | Lysosomal Disease Network |
|  | Society for Inherited Metabolic Disorders (SIMD) |
|  | Society for the Study of Inborn Errors of Metabolism (SSIEM) |
| Patient associations or nonprofit organizations | Bethanys Hope Foundation |
|  | Child Neurology Foundation |
|  | Cure MLD |
|  | European Leukodystrophies Association |
|  | MLD Foundation |
|  | United Leukodystrophy Foundation |
| Scientific conferences or annual meetings | ACMG Annual Clinical Genetics Meeting |
|  | ANA Annual Meeting |
|  | ACMG Annual Clinical Genetics Meeting |
|  | ANA Annual Meeting  CNS Annual Meeting  Congress of Neurological Surgeons |
|  | EAN Congress |
|  | EPNS Congress |
|  | EPNS Research Meeting in Paediatric Neurology |
|  | ERN-RND Annual Meeting |
|  | ESGCT Congress |
|  | ESHG Conference |
|  | European Conference on Rare Diseases |
|  | GLIA Scientific Conference |
|  | IGES Annual Meeting |
|  | International Conference on Genetic Disorders and Gene Therapy |
|  | International Conference on Human Genetics and Genetic Disorders |
|  | International Congress of Inborn Errors of Metabolism |
|  | ISPOR European, US and international conferences |
|  | SIMD Annual Meeting |
|  | SSIEM Annual Symposia |
|  | We’re Organizing Research on Lysosomal Diseases (WORLD)*Symposium*™ |
| Rare diseases databases | Orphanet |
|  | National Organization for Rare Diseases |

MLD, metachromatic leukodystrophy

Supplementary Table 3 Estimates of the number of live births per country included in review [55]

| Country | Number of live births (in calendar year 2023) |
| --- | --- |
| Europe | |
| *Czech Republic* | 99,736 |
| *Germany* | 755,129 |
| *Poland* | 423,741 |
| *Portugal* | 79,465 |
| *Sweden* | 113,685 |
| *The Netherlands* | 182,435 |
| *United Kingdom* | 977,708 |
| *North America* | |
| *Canada* | 379,615 |
| *Asia-Pacific* | |
| *Australia* | 301,714 |
| *Japan* | 813,751 |
| *South America* | |
| *Brazil* | 2,699,785 |
| *Middle East* | |
| *Turkey* | 1,214,053 |
| *United Arab Emirates* | 91,828 |
